# Letter to the editor: Re: Pathogenic mechanisms of osteogenesis imperfecta, evidence for classification

**DOI:** 10.1186/s13023-024-03269-9

**Published:** 2024-07-19

**Authors:** Raymond Dalgleish, Dimitra Micha, Andrea Superti-Furga, Fleur S. van Dijk, David O. Sillence

**Affiliations:** 1 Department of Genetics and Genome Biology, University of Leicester, Leicester, UK; 2 Department of Human Genetics, Amsterdam Reproduction and Development, Amsterdam Movement Sciences, Amsterdam UMC, Amsterdam, The Netherlands; 3 University of Lausanne and Genetica AG, Lausanne, Switzerland; 4 North West Thames Regional Genetics Service, London North West University Health Care NHS Trust, Harrow, UK; 5 Department of Metabolism, Digestion and Reproduction, Section of Genetics and Genomics, Imperial College London, London, UK; 6 Genomic Medicine and Paediatrics & Adolescent Health, Sydney University Clinical School, Children’s Hospital, Westmead, NSW Australia

**Keywords:** Osteogenesis imperfecta, Nosology, Classification

## Abstract

A paper published in Orphanet Journal of Rare Diseases proposes a new classification of osteogenesis imperfecta (OI) based upon underlying pathological mechanisms. The proposed numbering of OI types conflicts with the currently used numbering and is likely to lead to confusion. In addition, classification of OI according to underlying pathogenic mechanisms is not novel.

## Main text

A paper entitled “Pathogenic mechanisms of osteogenesis imperfecta, evidence for classification” [1] was published in this journal. The authors have attempted to bring order and consistency to the classification of osteogenesis imperfecta (OI) by creating a classification based on the “molecular pathogenic mechanisms of OI from the perspectives of type I collagen defects”. Any attempt to produce a classification that is understandable and practicable for clinicians and patients alike is commendable. Unfortunately, the classification presented in this paper is likely to lead to confusion because the authors have not taken account of major advances in OI classifications that have been published since 2010.

OI types have traditionally been numbered using Roman numerals since the 1970s as first suggested by Sillence and colleagues [2] who defined four types. Subsequently, numbering of OI types using Arabic numerals was introduced by the Nosology committee of the International Skeletal Dysplasia Society (ISDS) 2010 revision [3] which was expanded by van Dijk and Sillence in 2014 [4] who defined the five types and clarified the distinction between OI phenotype groups and the proposed severity grading. More recently, the 11th revision of the “Nosology of genetic skeletal disorders” [5] has introduced dyadic classifications for OI which retain the five OI types defined by van Dijk and Sillence accompanied by the HUGO Gene Nomenclature Committee (HGNC) gene symbol [6] for the gene that harbours the causative sequence variant. Each combination of OI phenotype and causative gene is assigned a nosology number, along with a descriptive name. For example, disorder number NOS 26–0010 corresponds to “Osteogenesis imperfecta, non-deforming (Sillence type 1), COL1A1 related”. This could usefully be abbreviated to “OI1-COL1A1”, although abbreviations of this type have not been formally approved by the nosology committee. The key issue to note is that Arabic numerals are used for each of the five OI types defined in the current nosology. As such, the phenotype and genotype are directly connected which provides immediate insight for patients, clinicians, and researchers.

The authors have not cited the three publications [3–5] that have adopted Arabic numerals for OI types, and they seem unaware that their proposed OI type numbers conflict with the numbering that is in current use. They define four OI types (1–4) which are based on the underlying pathogenic mechanism which can each include different genetic causes. These four types do not correspond to the widely accepted previously defined five clinical OI types (1–5), and do not take into consideration the many individuals and families with OI that have been diagnosed accordingly. This is likely to lead to confusion which is unhelpful for both individuals with OI and the clinicians involved in their care. If an individual is described as having OI Type 1, does this equate with the current phenotype-based non-deforming Sillence OI Type 1 or with the proposed pathogenic-mechanism based OI Type 1 which encompasses all clinical expressions of OI resulting from a defect of type I collagen, however caused?

In addition, classification of OI according to the underlying causative mechanism is not novel. There have been several published accounts in which the authors have grouped OI types according to their underlying mechanistic basis [7–9]. Indeed, one such account [7], cited by the authors, categorizes OI into five groups which are designated alphabetically (A–E) as compared with the four numeric categories that the authors now propose. Arguably, the previous alphabetic groupings are more useful as they categorise OI types in a more granular fashion without there being an unmanageable number of groups. The only slight deficiency is that there are now more OI genes than had been identified in 2016. We recognise that making sense of the myriad OI types is complex even for those with a long-standing professional interest in this disorder. To this end, one of us (DOS) has written a comprehensive explanatory account of OI and its underlying causes which is aimed at a general audience [10].

We would be grateful if the journal could review this paper in light of our concerns regarding the novelty of the authors classification proposals and the probability that confusion will arise if this new classification gains any level of acceptance.

## Data Availability

There are no data or materials to be made available.

## Declarations

### Authors’information

RD has investigated the molecular genetic basis of osteogenesis imperfecta and Ehlers Danlos syndrome since the 1980s. He established variant databases for these two disorders in the 1990s: https://lovd.nl/OI-genes and https://lovd.nl/EDS-genes.

DM has studied connective tissue disorders including osteogenesis imperfecta for many years. She now maintains the osteogenesis imperfecta variant database with colleagues at Amsterdam UMC.

AS-F is a clinical geneticist with specific interests in connective tissue disorders including osteogenesis imperfecta. He was corresponding author of both the 2010 and 2023 revisions of the nosology of genetic skeletal disorders.

FSvD is a clinical geneticist who has investigated osteogenesis imperfecta and Ehlers Danlos syndrome from both the clinical and genetical perspectives. She currently works at the North West Thames Regional Genetics Service, London UK, specialising in osteogenesis imperfecta and monogenic forms of Ehlers Danos syndrome. She is also on the Medical Advisory board of care4brittle bones. She co-wrote an extensive review of OI types with DOS in 2014.

DOS is a medical geneticist who established the first classification of osteogenesis imperfecta in 1979. He has contributed widely to revisions of skeletal disorder nosologies including the 2023 revision He currently serves on the International Nomenclature Committee for Constitutional Disorders of the Skeleton.

## Abbreviations

HGNC: HUGO Gene Nomenclature Committee
HGVS: Human Genome Variation Society
HUGO: Human Genome Organization
ISDS: International Skeletal Dysplasia Society
OI: Osteogenesis imperfecta
